# Development of deep learning chest X-ray model for cardiac dose prediction in left-sided breast cancer radiotherapy

**DOI:** 10.1038/s41598-022-16583-8

**Published:** 2022-08-12

**Authors:** Yutaro Koide, Takahiro Aoyama, Hidetoshi Shimizu, Tomoki Kitagawa, Risei Miyauchi, Hiroyuki Tachibana, Takeshi Kodaira

**Affiliations:** Department of Radiation Oncology, Aichi Cancer Center, Kanokoden 1-1, Chikusa-ku, Nagoya, Aichi Japan

**Keywords:** Breast cancer, Radiotherapy, Machine learning, Predictive medicine

## Abstract

Deep inspiration breath-hold (DIBH) is widely used to reduce the cardiac dose in left-sided breast cancer radiotherapy. This study aimed to develop a deep learning chest X-ray model for cardiac dose prediction to select patients with a potentially high risk of cardiac irradiation and need for DIBH radiotherapy. We used 103 pairs of anteroposterior and lateral chest X-ray data of left-sided breast cancer patients (training cohort: n = 59, validation cohort: n = 19, test cohort: n = 25). All patients underwent breast-conserving surgery followed by DIBH radiotherapy: the treatment plan consisted of three-dimensional, two opposing tangential radiation fields. The prescription dose of the planning target volume was 42.56 Gy in 16 fractions. A convolutional neural network-based regression model was developed to predict the mean heart dose (∆MHD) reduction between free-breathing (MHD_FB_) and DIBH. The model performance is evaluated as a binary classifier by setting the cutoff value of ∆MHD > 1 Gy. The patient characteristics were as follows: the median (IQR) age was 52 (47–61) years, MHD_FB_ was 1.75 (1.14–2.47) Gy, and ∆MHD was 1.00 (0.52–1.64) Gy. The classification performance of the developed model showed a sensitivity of 85.7%, specificity of 90.9%, a positive predictive value of 92.3%, a negative predictive value of 83.3%, and a diagnostic accuracy of 88.0%. The AUC value of the ROC curve was 0.864. The proposed model could predict ∆MHD in breast radiotherapy, suggesting the potential of a classifier in which patients are more desirable for DIBH.

## Introduction

Late cardiac toxicity after breast irradiation is a major adverse event in left-sided breast radiotherapy (RT)^1–6^. Darby et al. showed the relationship between the mean heart dose (MHD) and the frequency of major coronary events^5^. Deep inspiration breath-hold (DIBH) effectively reduces MHD compared to free-breathing (FB) RT^7–12^. Rochet et al. reported in their study that the reduction of MHD was > 0.9 Gy in 75% of patients and < 0.9 Gy in 25%^13^. Past studies have attempted to predict MHD using some parameters acquired in the simulation CT^14–27^. Most studies used such CT-based parameters, but some used non-CT parameters (e.g., BMI, pulmonary function test)^14,28–34^. Although non-CT parameters may have advantages over CT parameters in terms of earlier availability and reduced patient radiation exposure, no reports have high prediction accuracy using non-CT parameters. We previously investigated non-radiological parameters for preoperative prediction of MHD. Vital capacity was a significant predictor of MHD in DIBH (MHD_DIBH_)_,_ but it still did not work as an accurate prediction^34^.

The machine learning (ML) technique has been widely used in the medical field^35,36^. Many studies have used the ML approach with radiological images, and recently chest X-rays have been actively studied as a diagnostic ML tool in Covid-19^37,38^. Chest X-rays are the most frequently taken and easily available radiological images. Therefore, we wondered if the ML chest X-ray model could predict the cardiac dose of the breast RT, it might be easier and earlier to select which patients have significant benefit from DIBH.

The purpose of this study is to predict MHD in FB (MHD_FB_) and MHD reduction between DIBH and FB (∆MHD) using a machine learning method with preoperative chest X-rays.

## Methods

### Patient selection

This study is a prediction model development study approved by our institutional review board. All participants provided written informed consent and all methods were performed in accordance with the relevant guidelines and regulations. The eligibility criteria are as follows: histologically proven diagnosis of invasive ductal carcinoma or carcinoma in situ of the left breast, patients who underwent DIBH-RT after breast-conserving surgery from June 2018 to October 2021. Patients who did not receive preoperative chest X-rays were excluded. All data were retrospectively collected randomly split into two cohorts (training cohort: n = 78, test cohort: n = 25).

### Planning CT simulation

The DIBH-RT method of this study has implemented a technique of Bartlett et al.^10^. Described as our previous study, we trained patients to inhale, exhale, and hold deep breaths. The breath-hold training time was initially 5–10 s and increased to 20 s^26,34^. The simulation and training took about 20–30 min per patient. After confirming the respiratory motion, all patients underwent two planning CT simulations (FB and DIBH) in the supine position on a wing board with the arms stretched overhead. We used the Aquilion LB CT system (Canon Medical Systems, Tochigi, Japan) with a slice thickness of 3 mm.

### Treatment planning

We perform the contouring and planning on FB- and DIBH-CT using RayStation version 9 (RaySearch Laboratories AB, Stockholm, Sweden). The calculation algorithm is Collapsed Cone version 5.1. The planning target volume (PTV), including CTV with a 5-mm margin, was prescribed 42.56 Gy in 16 fractions with the Varian TrueBeam system (Varian Medical Systems, Palo Alto, USA)^26,34^. The clinical target volume (CTV) and the heart were delineated following the consensus guideline and atlas validation study^39,40^. The CTV was cropped withing 5 mm of the skin contour. Treatment plans consist of three-dimensional conformal radiotherapy using two opposing tangential beams and a field-in-field technique.

### Development of the chest X-ray model

**Figure 1:**
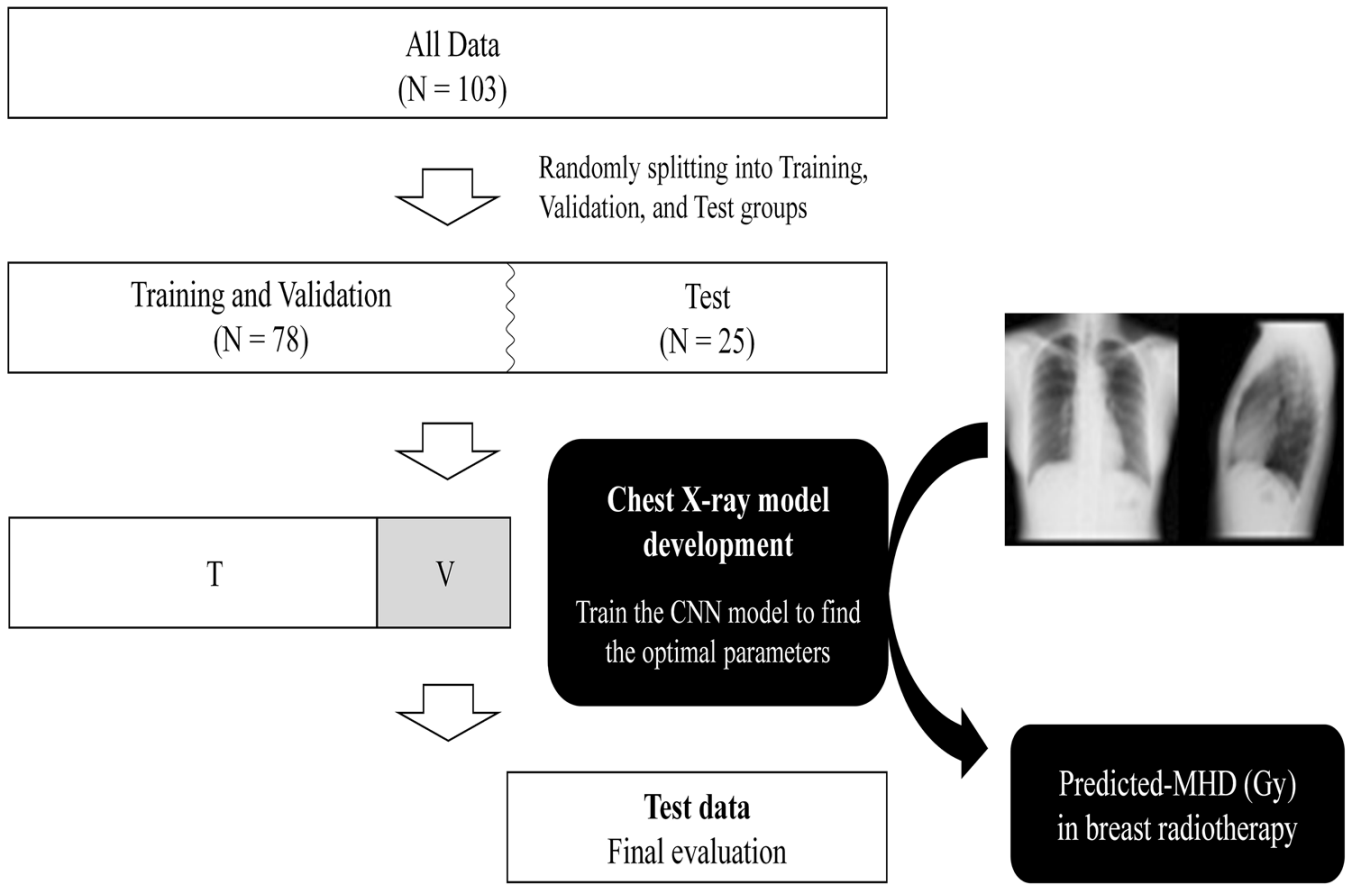
A pipeline of modeling procedure and model evaluation. *T* training, *V* validation, *CNN* convolutional neural network, *MHD* mean heart dose.

Figure 1 shows a pipeline outlining the modeling procedure and evaluation.

As the Transparent Reporting of a multivariable prediction model for Individual Prognosis Or Diagnosis (TRIPOD) guideline described, the data is split into the following groups; Model development group (Training: N = 59, Validation: N = 19), and Test group (Test: N = 25)^41^. Although the optimal ratio for the number of patients in each group has not been established, 60/20/20 and 70/15/15 are frequently used empirically; The ratio of each group in this study was determined based on several previous studies^27,42^. A regression model was trained with the training group, and the predicted MHD was validated against the validation group. Input values and size were searched from the parameters in previous studies and finally determined to achieve the best prediction results in the validation group^26,27,30,34,42^. Table 1 shows the convolutional neural network (CNN) architecture with the determined parameters.

**Table 1:** The detailed structure of CNN used in this study.

Layer	Output Shape	Connected to
Input1: $\text{X}1$ Input2:$\text{X}2$	1, 64, 64 1, 64, 64	
Mul ($\text{X}2\times \text{X}1$)	1, 64, 64	Input1, 2
Conv_1 Batch_norm_1 ReLU_1	16, 30, 30	Mul ($\text{X}2\times \text{X}1$)
Conv_2 Batch_norm_2 ReLU_2	16, 30, 30	ReLU_1
Dropout	16, 30, 30	ReLU_2
Full_connection_1 Batch_norm_3	100	Dropout
Full_connection_2	100	Batch_norm_3
Concatenate	103	Input3: $\text{X}3$ Full_connection_2
Full_connection_3 ReLU_3	100	Concatenate
Full_connection_4	1	ReLU_3

*CNN* convolutional neural network, *Mul* multiply, *Conv* convolution, *Batch_norm* batch normalization, *ReLU* rectified linear unit.

The architecture has three inputs: an anteroposterior chest X-ray image (1, 64, 64) as input 1, a lateral chest X-ray image (1, 64, 64) as input 2, and a patient's age (y), height (cm), and weight (kg) as input 3. First, we multiply the input1 and two tensors at the element level (i.e., multiplying each pixel of images). Then, convolution is performed twice for the multiplied data (1, 64, 64), followed by Rectified Linear Units (ReLU) and batch normalization. The resulting tensors were then fully connected and concatenated with input 3. Then performed another full-connection process, The predicted MHD was produced as an absolute value of the final output. Finally, predicted MHD is trained using the mean squared error as the loss function with 100 epochs.

### Model evaluation and statistical analyses

The primary prediction outcome is ∆MHD. The model is trained to achieve a high prediction accuracy of ∆MHD in the training cohort. The prediction performance of the developed model is evaluated in an independent test cohort. As our previous study^26^, we use the model as a binary classifier to determine if a patient would potentially receive ∆MHD > 1 Gy or not. The model performance is also evaluated as a regression model by calculating the median and interquartile range of absolute residuals, the coefficient of determination (R^2^), root mean squared error (RMSE), and mean absolute error (MAE). The secondary outcome is defined as the prediction accuracy of MHD_FB_. The prediction performance is evaluated in the same way as the primary outcome, but the cutoff value of classification is set as MHD_FB_ > 2 Gy following some previous reports^6,43,44^.

Statistical analysis was performed using R version 3.6.1 (The R Foundation for Statistical Computing, Vienna, Austria). The required sample size of test data is based on ∆MHD: we set the cutoff value of < 1 Gy as the classification point. According to our training data, 50% of patients had > 1 Gy. We estimated at least ten events (i.e., 20 patients) are required. *P* < 0.05 (two-sided) was considered statistically significant.

### Ethics approval and consent to participate

The Institutional Review Board (IRB) of Aichi Cancer Center Hospital approved our study (approve number: 2019-1-211).

## Results

### Dataset

One hundred and three patients were included in this study. Table 2 shows the patient characteristics of the training and test cohort. Each characteristic difference was not statistically significant between the cohorts. In the test cohort, median ∆MHD and MHD_FB_ were 1.24 (range 0.080–2.71) Gy and 1.97 (range 0.52–3.80) Gy, respectively. Fourteen patients (56%) had ∆MHD ≥ 1 Gy.

**Table 2:** Patient characteristics.

Characteristic	Training cohort (N = 78)	Test cohort (N = 25)
Age: median (IQR), years	52 (47–58)	58 (46–63)
Height: median (IQR), m	1.57 (1.54–1.60)	1.55 (1.52–1.61)
Weight: median (IQR), kg	54.0 (47.5–62.0)	51.3 (46.1–58.3)
The interval between chest X-ray and radiotherapy, median (IQR), days	82 (66–102)	104 (77–133)
**Tumor site**		
Inner-upper (A)	16	4
Inner-lower (B)	6	3
Outer-upper (C)	43	15
Outer-lower (D)	13	3
Center (E)	2	0
**TNM**		
Tis	12	2
T1N0	49	17
T2N0	10	3
T1–2N1	7	2
Other	0	1
**Molecular subtypes**		
Luminal (HR-positive and HER2 negative)	56	15
HER2 (HR negative and HER2 positive)	8	3
Luminal HER2 (HR and HER2 positive)	6	3
Triple-negative (HR and HER2 negative)	6	3
Unknown or other	2	1
Neoadjuvant chemotherapy, Y/N	13/65	6/19
**Surgery**		
BCS alone	2	2
BCS + SLNB (No ALND)	70	20
BCS + ALND	4	1
Other	2	2
Adjuvant chemotherapy, Y/N	7/71	3/22

*BCS* breast-conserving surgery, *SLNB* sentinel lymph node biopsy, *ALND* axillary lymph node dissection, *IQR* interquartile range.

### Model performance: MHD prediction results

**Figure 2:**
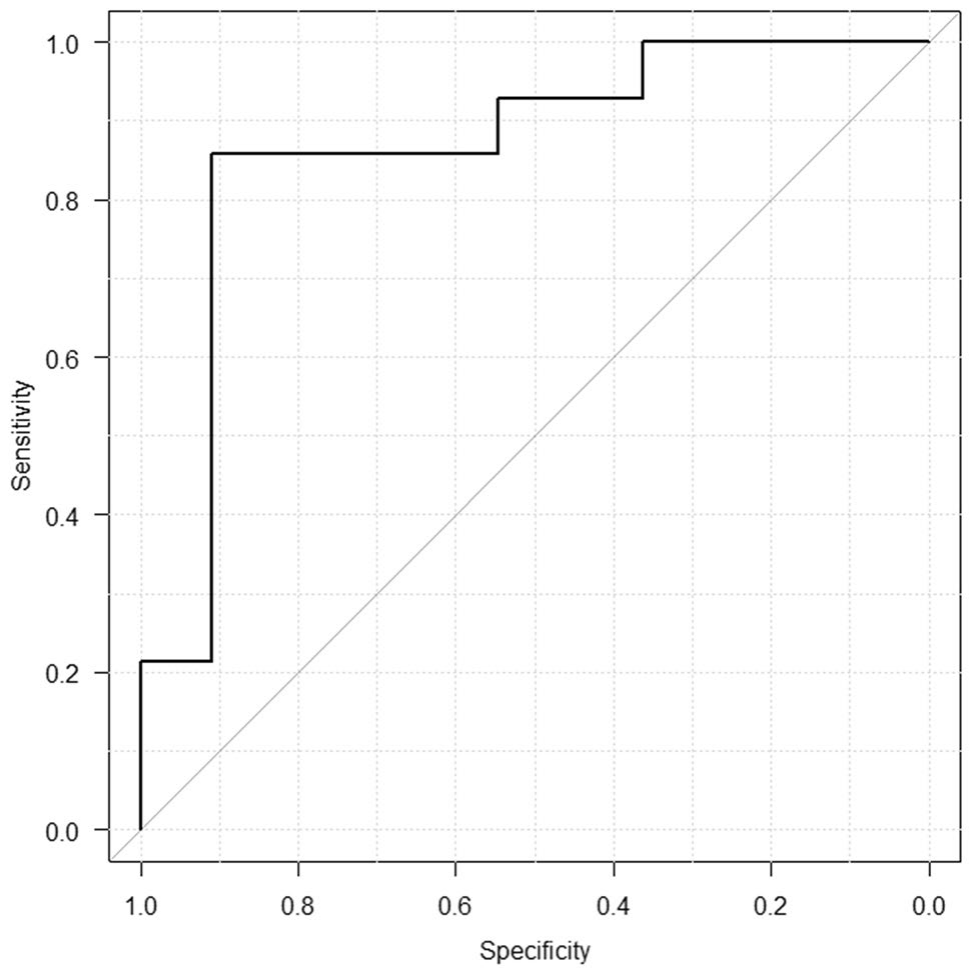
The Receiver Operating Characteristic (ROC) curve of the developed model: the area under curve (AUC) value was 0.864. The sensitivity and specificity of the best classification point (= 1.02 Gy) were 0.857 and 0.909, respectively.

As a binary classifier of ∆MHD > 1 Gy, the model showed a high classification performance: a sensitivity of 85.7%, a specificity of 90.9%, a positive predictive value of 92.3%, a negative predictive value of 83.3%, and diagnostic accuracy of 88.0%. Figure 2 shows the ROC curve, and the AUC value is 0.864 (95% CI 0.701–1.00). The point at 1.02 Gy was the best classification point in which the sum values of the sensitivity and specificity were maximized.

The developed model shows that the median predicted ∆MHD was 1.02 (range 0.06–2.43, IQR 0.63–2.11) Gy. Compared to the observed ∆MHD, the absolute prediction difference was 0.39 (range 0.004–1.55, IQR: 0.22–0.72) Gy. The Pearson correlation coefficient between observed and predicted ∆MHD was 0.55 (*P* = 0.028). R^2^, RMSE, and MAE were 0.30, 0.73, 0.56, respectively.

Although the accuracy was not as ΔMHD, MHDFB could also be predicted from the model: the median absolute error was 0.72 Gy (range 0.058–2.73 Gy, IQR 0.43–1.42 Gy), the correlation coefficient was 0.46 (P = 0.02), and the sensitivity and specificity were 0.58 and 0.77, respectively.

## Discussion

Recent studies have attempted to predict MHD to select patients with potential cardiac toxicity risks and reduce MHD by performing DIBH^14–26^. In most cases, prediction models used the maximum heart distance or cardiac contact distance in the CT simulations as predictors^14–20,24^. The coronary artery calcium scores (CAC) in CT improved the Framingham risk score prediction for coronary artery disease (CAD)^45,46^. According to Mast et al., DIBH increases LAD CAC less than FB, potentially preventing radiation-induced coronary artery disease^47^. Our previous study demonstrated that a synthetic DIBH-CT model with a deep learning approach achieved more accurate ΔMHD prediction than other models^26^. However, such models in past studies have a significant limitation: the prediction is only performed after simulation CT.

We next investigated non-radiological parameters for preoperative prediction of MHD^34^. The result showed that Vital capacity was the only significant predictor of MHD_DIBH,_ but it could not work as a predictor of ΔMHD nor MHD_FB_ as other parameters. To the best of our knowledge, no other studies have found non-CT parameters promising as predictors of ΔMHD nor MHD_FB_. Therefore, this study attempted to predict ΔMHD nor MHD_FB_ using a deep learning technique based on preoperative chest X-rays. The prediction results showed a high performance as a binary classifier in the cutoff of ΔMHD > 1 Gy. Our model has also worked for MHD_FB_ prediction in the same method. The strong points of this model are the early timing of the prediction and the required radiological images required only chest X-rays, which can be acquired easier and earlier than simulation CT in many patients. Ninety-two percent of our patients underwent preoperative chest X-rays, with a median of 90 days before radiotherapy.

In the present study, MHD_FB_ and ΔMHD were used as predictive outcomes, following previous studies^14,26,28–34^. The primary outcome was defined as ΔMHD, used in multiple studies^14,26,30–33^. We set the cutoff for classification as ΔMHD > 1 Gy based on the report of increased cardiotoxicity per 1 Gy by Darby et al.: a linear relationship between MHD and the frequency of major coronary events that increases at a rate of 7.4% per Gy, but no significant difference was found for MHD < 2 Gy^5^. Otherwise, the Early Breast Cancer Trialists’ Collaborative Group report and the UK consensus statements for postoperative breast radiotherapy recommend the MHD < 2 Gy, so it may be possible to set the classification criteria with MHD_FB_ as the primary predictive outcome^6,43,44^.

There are several limitations of this study. First, our study used a single institutional dataset, consisting mainly of those who underwent BCS followed by DIBH-RT. Therefore, whether the study results can be extrapolated to patients undergoing chest wall or lymph node irradiation is uncertain. Second, our approach focused on the chest X-ray parameters and may omit the clinical aspects of DIBH training during simulation: even if the prediction recommends the cardiac sparing RT, our model does not predict whether the patient can tolerate DIBH. Finally, the CNN architecture used in this study requires both anteroposterior and lateral chest X-ray images. Future studies are needed to build a model using only anteroposterior images and perform external validation at multicenter for model versatility.

## Conclusion

In conclusion, our deep learning chest X-ray model can predict MHD and play an essential role in classifying patients’ potentially desirable DIBH. However, further study is needed to validate our prediction model externally.

## Data Availability

Research data are stored in an institutional repository and anonymized numerical data will be shared upon request to the corresponding author. Research image data are not available at this time.

## Abbreviations

DIBH: Deep inspiration breath-hold
MHD: Mean heart dose
FB: Free-breathing
RT: Radiotherapy
BMI: Body mass index
MHD_FB_: MHD in FB
MHD_DIBH_: MHD in DIBH
ML: Machine learning
CTV: Clinical target volume
PTV: Planning target volume
MLC: Multileaf collimator
CNN: Convolutional neural network
ReLU: Rectified linear units
R^2^: Coefficient of determination
RMSE: Root mean squared error
MAE: Mean absolute error
